# Diseases of the Uterus

**Published:** 1837

**Authors:** 


### Art. VI.—Diseases of the Uterus.

*A Treatise on the Functional and Organic Diseases of the Uterus.* From the French of F. Duparcque, Docteur in Medicine de la Faculte, &c. &c. Paris. Translated, with notes, by Joseph Warrington, M. D., of Philadelphia. 1837. 8vo. pp. 455.

In entering upon an analysis of the work whose title is prefixed to the present article, it may not be amiss to apprize the readers of our journal, that it emanates from a country where the restrictions imposed in our own upon the investigation of uterine diseases, by female delicacy, (a delicacy which, however much it may interfere with the accuracy of our disagnosis, we are nevertheless constrained to respect,) are almost unknown, or at least, are rendered inoperative ; and where, too, the greatest facilities are enjoyed for testing the correctness of pathological observations upon the living subject, by unrestricted dissections of the dead. The work is moreover the production of an eminent and practical physician, whose knowledge and experience in this department of obstetrical practice, as embodied in the volume before us, obtained for him the prize offered by the Royal Society of Medicine, of Bordeaux, and at the same time its high commendation.

Ushered into the world under circumstances so auspicious and so flattering, we could not hesitate to give to the treatise the tribute of a few pages, even though in our judgement it did not fully realize the exalted expectations excited by the splendor of its *accouchement.* But, besides these considerations, which are in themselves sufficient to arrest the attention of the professional reader, and to constitute our apology for this intrusion upon his time, we are not without other motives to animate us in the task we have undertaken. Among these, we may be permitted to advert, as the most influential, to the hope we entertain that our labor may be the means of awakening Western physicians to a more diligent and discriminating study of a class of affections which, regarding their immense influence upon human happiness and human life, are most shamefully and criminally neglected in our country.

The work is divided into two parts. The first is devoted to a cursory examination into the origin, predisposing and determining causes of "chronic alterations" of the uterus—their mode of production and developement—their respective degrees of curability—and the means of ascertaining these alterations. The second part treats more particularly of the chronic diseases of the uterus, which comprehended by our author, under the general terms of engorgement and ulceration, are divided into a variety of species ; a classification which, however, we need not further pursue at this time, since in the progress of our analysis, we shall have occasion to recur to its most minute subdivisions, and under each to indulge in such explanatory and critical remarks, as may seem to us proper for enforcing truth or correcting error.

M. Duparcque sets out with the general proposition that the uterus is not "very susceptible of diseases, either acute or chronic before puberty." This want of susceptibility to morbid action is readily explained, by the disproportionate and unequal development of the organ in infancy and adolescence, compared with other parts of the animal economy ; by its feeble vital endowment ; by its isolated condition, neither receiving from, nor exerting upon the other organic apparatus of the system any other than a very feeble influence ; and lastly, by its anatomical position which exempts it from the operation of mechanical, physical, and chemical agents." But if these causes give to the uterus a remarkable immunity from disease in early life, the physiological changes which take place about puberty, expose it to a multitude of morbid actions, whose bitter fruits, however, are sometimes not experienced, indeed seldom are, till the woman has passed the grand climacteric of female existence, and is descending into the vale of life. Not to say any thing of the great augmentation of the vital powers of the uterus at puberty, of its increased sensibility, or of its numerous sympathetic relations, as sources of disease, we need only recur to the state of the circulation in the organ at this time, to enable us to comprehend at once how many of the uterine affections of early life, such as congestions and inflammations, are induced, and the foundations laid for more serious and graver maladies, in subsequent existence. On this subject our author remarks—

"In the early periods of puberty, the uterus is not always in a condition to emit the fluids that give rise to the menstrual movement in its tissue. In consequence of which, a local plethora or congestion occurs, manifested by a sense of heaviness in the hypogastrium, more or less severe pains, denominated uterine colic or uterine tenesmus, alternate chills and flushes, head-ache, difficult respiration, and sometimes hysteric fits.

This fluxionary movement having existed a few hours or days, subsides spontaneously without any discharge; it re-appears with the same symptoms at the following menstrual period, until the exhalent extremities of the vessels becoming more permeable, readily disgorge themselves.

In some girls, however, this state of things continues through an indefinite period; the congestion of the uterus does not completely pass off after each period, but becomes increased, the local and general disturbance acquires intensity, and medical aid is required to prevent the dangerous consequences of this morbid state.

The engorgements of the uterus may under these circumstances, take on the form of simple congestion, of acute, and particularly chronic inflammation, which may successively or simultaneously pass through all the stages of suppuration, or cartilaginous or osseous degenerations. Restricted in the degree of its action, this engorgement becomes the basis of cancerous formations in the course of some years."

As illustrative of these general views in respect to the origin of organic affections of the uterus, our author furnishes many cases, of which, however, it does not comport with the limits of a review to speak in detail.

The first is a case of emansio mensium, occuring in the person of a young lady, aged 15 years. Exhibiting the "exterior signs of puberty, the phenomena which prelude the catamenial discharge, were manifested for some months at irregular periods, but the secretion did not take place." After a persistance of this state of things, the patient was at length, at the ninth period, seized with the symptoms of acute mitritis; but these were readily subdued by the prompt employment of an active antiphlogistic treatment, which, however, was not effectual in establishing the function of the uterus. At each menstrual discharge, the girl continued to be harassed by pains in the loins and hypogastrium, which gradually becoming worse, were at length accompanied by oppressed respiration, palpitations, and occasionally by catalepsy. "Bleeding had been found to be the most useful remedy in preventing the increasing violence of the paroxysms."

Under the impression that marriage would correct "this morbid condition of the uterus," the young lady entered into the connubial state at the age of twenty; but instead of deriving the anticipated relief from this measure, the painful phenomena were all aggravated. At length, three years after the event to which we have adverted, our author was called to attend her.

"At this time, she could not stand erect, in consequence of the severity of pain in the loins, hips, groins, and the anterior part of the thighs. She was afflicted with the dyspnœa and cephalalgia; the pulse which beat 110, was hard and contracted; her countenance was flushed.

An examination per vaginam, enabled me to discover that the neck of the uterus was short, thick, and confounded with the body of the uterus, which could be felt through the vagina, in passing the finger around the neck; the uterus appeared to be as large as at two months of pregnancy; the mouth of the uterus was partially distended, and filled with a viscid matter. I could seize the fundus by applying the left hand above the pubes, and pushing the abdominal parieties towards the sacrum; it was regular in shape, and about the size of a goose egg. The pain was greatly increased by this examination, and the patient was seized with a paroxysm of hysteria."

The case thus detailed is instructive in several points of view. In the first place, it demonstrates, along with others which are related by M. Duparcque, that virgins, as well as matrons, are occasionally the subjects of organic alterations of the uterus, though, as he subsequently remarks—"these changes, in the former case, affect the entire substance of the organ, while, in the latter, they are for the most part confined to the mouth and neck of the uterus."

In the second place, we are admonished of the error of regarding amenorrhœa as an idiopathic disease dependent upon uterine debility, and requiring local stimulants for its removal. As, in the instance before us, it is usually but the symptom of a pre-existing malady, and until we learn to discriminate its causes, and to detect the associate states, whether of the system or of the organ, our practice will be empirical, and, to say the most of it, attended by an equal chance of failure as of success. Menstruation may be considered as consisting essentially of two parts;—a fluxionary movement, by which blood is determined to the uterus; and an exhalent or secretory process, by virtue of which the menstrual fluid is elaborated. Now it is evident that the healthy performance of the menstrual function depends upon a due correspondence or relation between these two movements; and hence, when amenorrhœa is associated with acute or chronic engorgement of the womb, we can no more expect to produce a healthy catamenial flux by means which determine blood to the uterus, than we can hope to produce the elaboration of a healthy chyme by accumulating food in a crippled stomach. As in the latter case, the cure must proceed upon the abstraction of nutrient materials to the reduced powers of the stomach; so in the former, relief must be sought by the judicious employment of all the means which are calculated to reduce the sanguine remora of the uterus. In these cases, instead of endeavoring to goad on the organ by indulgence in venery, or by other local excitants, which, under the denomination of emmenagogues, are presumed to exert a special action upon the genital apparatus, we should place our reliance upon a systematic and persevering antiphlogistic course, proportioned to the intensity of the congestion. Blood-letting, general or topical, cathartics, antimonials, baths and emollient fomentations, are the appropriate remedies; and of their efficacy, under the circumstances stated, many of the cases of our author furnish the most irrefragable proof.

To detail these, we repeat, forms no part of our plan; but M. Duparcque's fourth case is so remarkable in many respects, that a condensed statement cannot but prove interesting. A girl, aged 15 years, had menstruated regularly for several months. But, at each period, she suffered excruciating pains in the hypogastrium, antecedent to the flow, which always brought relief, and was generally abundant for five or six days. In this state, she was suddenly alarmed "by an explosion in an adjoining chamber; an icy coldness pervaded her body, and was soon followed by violent agitation," pains in the abdomen, and suppression of the menses. A recurrence of her period brought with it additional suffering, but no return of her catamenia. Her health declined. Medical advice was obtained, but her symptoms becoming daily more alarming, M. Duparcque was requested to see her in consultation. Upon examining the abdomen, a tumor was discovered in the hypogastric region, behind the pubis, but somewhat inclined towards the right Iliac fossa. This tumour resembled in its position, form, and volume, the gravid uterus at three and a half months, and was very hard and sensitive, so much so, that the slighest pressure upon it was painful. "In the left Iliac region, there was another oblong nipple-like tumor, dipping deeply into the pelvis. Pregnancy was suspected, a suspicion which seemed to derive some confirmation from the reluctance of the patient to submit to an examination. It being difficult to pass the finger into the vagina, an attempt was made to explore the uterus through the rectum; but this, too, failed, in consequence of the intestine being blocked up with indurated fœces. With these obstacles to an accurate investigation of her case, the patient presented the symptoms of synochal fever. Sixteen ounces of blood were abstracted from the arm, and the fœcal matters were removed from the rectum by the handle of a spoon, after which copious dejections were procured by castor oil.

On the next day, the abdomen was soft, and the tumor in the left Iliac region had disappeared, whilst that in the hypogastrium had not diminished in volume, but had in some degree changed its relations by falling more under the linea alba, and sinking into the pelvis.

The introduction of the finger into the rectum, and the application of the other hand upon the hypogastrium, satisfied M. Duparcque that this tumor could be no other than an enlarged uterus. The question now arose, as to the cause of this enlargement. There were many, and almost insuperable objections to the presumption of pregnancy; and in the fact that she had menstruated regularly, was found positive demonstration that the augmentation could not be owing to retained menses, in consequence of occlusion of the os tincœ, or an imperforate state of the hymen. The inference was therefore strong, that the tumor arose from an engorged state of the uterine tissue, which, though partaking at first of the character of chronic, had now all the marks of acute inflammation—such as pain and great sensibility under pressure.

Guided by this pathological view, the patient was ordered to lose 12 ounces of blood from the arm; next day 20 leeches to the lower abdomen, followed by emollient cataplasms, which, with baths and laxatives, were continued for four days. At this time the tumor was very much reduced, and the condition of the patient altogether improved, being free from nausea and fever, and almost from pain—twelve leeches and continuance of the other remedies with very light fluid diet.

At the expiration of ten days more, the tumor could scarcely be felt. The menses soon after made their appearance, and the patient's health was perfectly re-established.

We have selected the above case, not as exemplifying the treatment alone, but as combining several peculiarities of interest. The profession has been long familiar with the fact, that pregnancy is often simulated by distension of the uterus, in consequence of the confinement within its cavity of menstrual blood, but that the organ in the virgin state ever attains such an increased volume, by simple sanguine engorgement of its tissue, as to perplex the diagnosis, is not, we think, generally understood by medical men; and M. Duparcque, in bringing the fact to their notice, and thus revealing another reason against hasty and inconsiderate deductions to the prejudice of female character in a most sensitive point, deserves to be esteemed as a benefactor to science and to humanity. There is still another peculiarity in the case, which strongly illustrates the duty of careful, diligent and patient inquiry in all our pathological investigations. The accumulation of fœcal matter in the sigmoid flexure of the colon, was a circumstance well calculated to embarrass the diagnosis of an inexperienced practitioner; and if it did not perplex the judgment of our author, it was owing to his enlarged experience, inculcating a lesson of caution, which, though inscribed on every page of medical history, is seldom acquired except through disastrous mistakes.

M. Duparcque avails himself of the two succeeding cases to dwell at some length upon the etiology of uterine congestions. In his view, coition, "by its abuse or by its use in certain conditions of the female system," may create or perpetuate uterine engorgements, of which "the only appreciable phenomena are derangements of the *menstrual function*," and sterility. In the treatment of this state, it often happens that the mind of the medical attendant, being pre-occupied with the resulting phenomena, overlooks the primary derangement; and his efforts to reinstate the uterus in its functions, are limited to the employment of emmenagogues and local stimulants—a practice which too surely confirms the maladies it is intended to relieve, and lays the foundation for the developement, in the progress of time, of more profound organic alterations. In these cases, the only rational practice consists in a "complete abstinence from sexual intercourse, in the adoption of a mild regimen and anti-phlogistic means." A rigid perseverance in this course, aided by whatever may contribute to an equilibrium of the functions of the system, and thus diminish indirectly abnormal excitement in the uterine apparatus, will very generally resolve its congestion, remove the amenorrhœa or dysmenorrhœa dependent upon it, and restore to the organ the power of conception.

It is the same neglect to refer diseases to their ultimate causes, which has led physicians to recommend matrimony for the cure of hysteria. This is an affection, which every day's experience assures us, our predecessors were right (very generally at least) in referring to a uterine origin. It may be the result of functional disorder, (a view too generally adopted,) or of an altered condition of the organ associated with engorgement or sanguine excitement. When dependent upon the latter cause, the excitation of the genital organs by connubial intercourse or stimulating emmenagogues, is mischievous, and founded upon rank empiricism. Of the correctness of these pathological and therapeutic views, the sixth case of our author furnishes indubitable and almost conclusive evidence.

But to return from this digression, we conclude with our author, that "masturbation" or coition, "by the repeated and permanent excitation which it awakens in the generative organs, makes them a centre of determinations, and thus may cause chronic engorgements, manifested only by "menstrual disorder," deprive the uterus of its power of conception, and lay the foundation for the more tardy appearance of profound organic alterations." But it is not by excitement or mechanical irritation alone, that coition becomes the cause of uterine affections. The venereal virus which usually expends its force upon the external organs, "sometimes immediately attacks the neck of the uterus," and developes there various organic changes, "such as engorgements, ulcerations and vegetations."

Fruitful, however, as is this source of uterine disease in married life, conception in all its consequences, according to M. Duparcque, is a much more prolific cause.

"The organization and vitality of the uterus are susceptible of new modifications. Its tissue becomes expanded and more permeable to the increased determination of fluids into it; the exhalent orifices are devloped to establish a more ample communication between the uterine vessels and that of the placenta; and lastly, the organ acquires the power of rapidly contracting itself.

"Although the separation of the parieties of the uterus for the developement of the product of conception, depends upon a sort of inherent expansibility, it is not always readily disposed to effect this extension; this indisposition, occasions in the course of the gestation, bearing down or dragging pain, and disagreeable tension, which are soon followed by abortion. Young ladies are most exposed to this accident, and we are assured that the greatest number of abortions or miscarriages occur in first pregnancies. It frequently happens that the first abortion is succeeded for a long time, or perpetually, by a difficulty in menstruation and a consecutive sterility. Numerous cases have proved to us that these functional disturbances result from a chronic inflammatory engorgement of the uterus or of its neck only, which is susceptible of cure. Some cases of uterine cancer, not observed till after the cessation of the menses, have proved to us, that these diseases took their origin in a first and only abortion, after which the menstrual function had been disordered, and the woman subjected to more or less severe, protracted and constant pains in the loins, etc.; all—symptoms denoting a morbid state of the uterus.

"Indulgence in copulation, and the neglect of hygienic and therapeutic precautions after abortion, doubtless contribute in no small degree, to maintain the chronic inflammation, occasioned both by the distention of the uterus beyond its intrinsic expansibility, and by the painful contractions brought on for the purpose of expelling the product of conception.

"Premature delivery, when it has been laborious, is a much more prolific cause of acute, though more frequently chronic affections of the uterus, than parturition occurring at full time. The neck of the uterus first feels the effect of these lesions; it is also the part of the organ most apt to be affected in the lying-in, in consequence of the pressure it sustains between the head of the infant and edge of the superior strait, the violent and forced distention in a too rapid labor, by imprudent manœuvres, as the introduction of the hand for the purpose of turning the child, or the application of instruments, causing contusion and laceration of the sides of the orifice.

"The violent application of the parieties of the uterus upon the body of the infant, when the contractions are very energetic, especially when the liquor amnii has been prematurely discharged, the irritation from turning the child in the cavity, the forcible extraction of the placenta, before its separation from the sides of the uterus; the manœuvres of the hand in an attempt to detach and extract it, the mechanical and medical means employed to excite the contractions of the uterus when in a state of inertia, and to arrest hemorrhage, etc., are so many causes, capable of irritating the uterus, and exciting congestions, inflammations or engorgements, either in the whole, or a part of the body of this organ: these consequences more readily arise from the persistance of the humoral and vital determinations which are necessary to the development of the product of conception. This congestive action bearing a strong resemblance to the menstrual molimen, may be much more easily disturbed by similar causes, whence analogous but more intense morbid effects result. In fact, every thing capable of arresting the discharge of the lochia, without arresting the determination which furnishes the materials, becomes the cause of congestive or inflammatory engorgement of the uterus. Such are the impressions of cold, the rigors of an intermittent, and moral emotions of various characters."

As an occasional effect of the engorgement of the uterus consequent upon delivery, our author takes a passing notice of prolapsus uteri, and delivers a very important caution in regard to the use of pessaries in the congested condition of the organ; a caution which we should have supposed, was unnecessary, had we not been informed by him that "he had frequently seen the unfortunate mistake committed by physicians of celebrity. In our country, we venture to say, there is no student of medicine who had attended a single course of lectures, but would blush at the presumption of such ignorance, and certain we are that there is no patient who would submit to the excruciating tortures of such practice. Emollients, resolvents and rest, constitute the only rational treatment of such a case. In regard to the opinion combated by M. Duparcque, that pregnancy effectually cures prolapsus of the uterus, we are not prepared to assert that it is maintained, by any respectable authority within our reach, in the unqualified manner in which he announces it. But that it often affords temporary relief, is a very general sentiment in the profession, and is explicable upon a very obvious and intelligible principle. Coincident with this opinion, is our own experience.

Of the power of the *misnamed* "critical age" over the female economy, we have long thought that erroneous and exaggerated notions were entertained and promulgated by many medical writers; and, in this opinion, we are happy to have the distinguished support of M. Duparcque. That the fears entertained by women in regard to the cessation of the catamania are more imaginary than real, is sufficiently manifest to our mind from an inspection of the statistical tables of Moret, Finlaison and Chateauneuf, which prove that as many men as women die between 40 and 50 years of age.

"It is improper," says the author, "that we should indiscriminately ascribe to the critical age, all the diseases which are manifested at this time. It should be borne in mind, that a great many of the alterations considered as resulting from the cessation of the catamenia, have originated at an anterior period; the " turn of life," merely developes these affections, changes their character, or accelerates the successive transformations of which they are susceptible.

"In a very exact history taken from 40 women, between the ages of 40 and 50 years, who were affected with cancer of the uterus, only five were found in which the disease was of recent origin, or had resulted from the critical period; in 33 others, the catemenia had exhibited some irregularities since their last confinement, or after an abortion, or in consequence of one of whatever causes we have seen capable of exciting them in women, which the dysmenorrhœa and the constant sterility, and other symptoms developed in the region of the pelvis, indicated some alteration in the uterus, which had immediately succeeded accouchment, abortion, or an accident,"

And finally, in two cases which are cursorily narrated, the affection appears to have originated "about the period of puberty."

"It may therefore be established as a general position, that the critical age is dangerous only to those women who arrive at this period with an alteration in the uterus already existing, and originating at a time more or less remotely anterior.

"When the critical epoch has passed without accident, or when those diseases which were at that time manifested, have yielded either to time or to appropriate remedies, the constitution of the woman seems to approach that of the man, she acquires organic force and vital resistance. The uterus has then completely returned to its primitive inertia, and the woman experiences an exemption from all diseases which are not common to the other sex. With very few exceptions, the uterus is no more subject to organic changes, than any other portion of the system.

"Those organic alterations which are developed in the uterus in aged women—or which having been developed at a period more or less anterior, and have not received an unfavorable impulse from the critical period, are slow in their progress; and notwithstanding the frequently considerable organic disorders which constitute them, they do not exert any conspicuous action on the general health. The uterus in discarding its functions, has at the same time lost the influence which it exercised over all the economy; and the diseases which afflicted it, do not radiate to any other parts."

In confirmation of the doctrine contained in the last paragraph, two cases of severe organic disease of the uterus are related, in which there was not only no symptom to indicate the existence of such affection, but no material derangment of the general health. The subjects of each passed through a long life, and ultimately died of other disease.

M. Duparcque next treats of the formation, development and termination, of "the organic alterations" of the uterus. The sanguine engorgements, in common with every other person, he considers to have their primitive seat in the capillary vessels of the womb, and so long as the blood is confined within the vascular tubes, they are susceptible of *resolution;* and this he regards as a practicable event, even when the blood has transcended its healthy limits, and has exuded into the parenchyma of the organ, "provided the original structure maintains its integrity." "When the tissues are destroyed, or profoundly altered as in inflammation with carnification in the highest degree of congestion, a reduction to the normal state is impossible."

Two elements enter into the composition of all indurated schirrous and cerebreform productions; the one an organized cellular net-work, the other a matter deposited in its meshes. The first, or cellular, is the original elementary tissue common to all "organic alterations," and it is for this reason that schirrus, fungus hematodes and melœna, present the same individual but distinctive characters, no matter in what organ or tissue of the body they happen to be located, whether "in the skin or subcutaneous cellular structure, in the stomach or the epiploon, the liver, lungs, spleen, or uterus." The second element or adventitious matter is the product of an "abnormal vital elaboration." It does not exist in the blood already formed, but is fashioned, as it were, from this fluid, by a process similar to that which produces the normal, or healthy tissues of the economy." The natural growth of the body and the development of its parts, hypertrophy and schirrus are equally the product of a secretory action; with this difference, however, that in the latter disease, as well as in all others formed by the deposition of heterogeneous matter, the vitality of the secreting apparatus is so altered that it is no longer capable of selecting from the blood the elements proper to itself, but only those, "the combination of which constitutes the morbid growth."

By some pathologists, this alteration in the secerning tissue is presumed to partake of the character of inflammation, but of inflammation changed and modified by peculiarity of the constitution or of the part. In favor of this hypothesis, it is alledged, that the causes which give rise to the development of the accidental tissues under review, are the same which excite inflammation, and that the remedies which have been found most effectual in their treatment, are also such as subdue vascular action. Notwithstanding, however, the speciousness of this reasoning, it must be admitted that these morbid depositions do occasionally take place without previous inflammation, and in the midst of healthy structures without the operation of any of those causes which produce the latter disease. But whatever is the character of this peculiar vital modification, it is certain that it displays itself chiefly in middle age. Advanced life overcomes it ; and therefore—

"We very rarely see cancerous affections of the uterus originate in old women ; the cancerous diathesis is more rare at that time of life, and when these alterations, developed at an anterior period of life, have passed over a certain space of time without destroying the patient, we sometimes see them diminish, progress more slowly, or else pass through all the local degenerations without materially changing the health of the women who are affected by them."

These views and the results of operations for cancers practised for a number of years in the Parisian hospitals, going to show that relapses are less common in proportion as the patient is advanced in life, have led our author

"To conclude, that far from urging to the early practice of the operation, it would be better to wait until the disease had, so to speak, spent its action upon the impaired organ. In delaying the operation, we permit the patient to acquire the age in which the vital or organic modifications, which constitute the predisposing essential conditions of the cancerous alterations, change and dissappear, and the disease henceforth confined to the part which it had attacked, will not be subject to relapse."

Of the soundness of this advice, we are very dubious, and until it is sustained by multiplied facts and enlarged experience, we prefer to rest upon the ancient canons of the profession, and to recommend early extirpation of all cancerous tumors.

[To be continued.]

